# The First New Zealanders? An Alternative Interpretation of Stable Isotope Data from Wairau Bar, New Zealand

**DOI:** 10.1371/journal.pone.0135214

**Published:** 2015-10-28

**Authors:** Andrew A. Brown, Tim Thomas

**Affiliations:** 1 Institute of Archaeology, University College London, London, United Kingdom; 2 Department of Anthropology and Archaeology, University of Otago, Dunedin, New Zealand; University of South Carolina, UNITED STATES

## Abstract

*PLOS ONE* Volume 8 includes an article “The First New Zealanders: Patterns of Diet and Mobility Revealed through Isotope Analysis”. The paper proposes that burial groups within the settlement phase site of Wairau Bar differ in terms of dietary stable isotopes and ^87^Sr/^86^Sr. The authors argue this difference is probably due to one group being a founding population while the other burials are later. Here we review the work of Kinaston *et al*. and present an alternative analysis and interpretation of the isotopic data. Treating the isotope data independently from cultural and biological factors we find that sex best explains dietary variation. Our reassessment of ^87^Sr/^86^Sr confirms the authors original finding of high mobility of early New Zealanders but suggests a larger range of individuals should be considered ‘non-local’ on current evidence.

## Introduction

The manner of Polynesian adaptation to, and exploitation of, the New Zealand environment in the decades following colonisation is a central focus for archaeological research in the region. Some of the most important and far-reaching questions archaeologists have asked are also the most basic: what were people eating and how mobile were early groups? The answers to these questions have important implications for a broader understanding of settlement, seasonality, socio-political complexity and culture change in New Zealand. Diet and mobility reconstructions therefore remain an important component of New Zealand archaeology [1, 2, 3]. Here, we consider a reconstruction of human diet and mobility based on stable isotopes presented in the recent paper by Kinaston *et al*. [4] entitled “The First New Zealanders: Patterns of Diet and Mobility Revealed through Isotope Analysis”. The study is based on *koiwi tangata* (human remains) from Wairau Bar, a large settlement occupied early in New Zealand prehistory, ca. AD 1300 [5, 6]. Wairau Bar contains a rich array of early material culture unmatched in New Zealand archaeology. The abundance and range of artefacts has led to the site being considered the type-site of the East Polynesian Archaic phase in New Zealand [5, 7]. Throughout New Zealand and East Polynesia the ‘Archaic’ is associated with colonising populations and, as such, represents the culture of the first generation of settlers in the region.

As the first large-scale isotopic analysis of human remains in New Zealand, and one of the few in East Polynesia, the analysis by Kinaston *et al*. represents a significant advance in refining our understanding of diet and mobility in the region. The authors model diet using carbon (δ^13^C) and nitrogen (δ^13^N) stable isotope ratios in human bone collagen and analyse mobility through strontium isotopes (^87^Sr/^86^Sr) in tooth enamel. Traditional reconstructions of diet using faunal remains and mobility using proxies such as lithic exchange present aggregate or population level behaviours. However, the analysis of stable isotopes affords the opportunity to assess diet and mobility at an individual level, and thus identify sub-groups or outliers in a population. Kinaston *et al*. ([4]: 8–9) argue that isotopic analysis of the skeletons from Wairau Bar reveals distinctions between sub-groups within the cemetery population and that, tantalisingly, one group (Group 1) may represent an immigrant colonising population at the site and, by extension, New Zealand. While we strongly support the attempt to reveal patterns of diet and mobility through isotopic analysis, we believe the authors interpretations are constrained by the focus on a single explanatory variable and by the use of *a priori* divisions in the analysed sample. Ultimately, these divisions introduce bias, which leads to conclusions that we feel are not supported by the available data. Here we review the work of Kinaston *et al*. [4] and offer an alternative interpretation of the isotopic patterns at Wairau Bar.

## Critical Review of Isotopic Analysis from Wairau Bar

Central to the analysis of Kinaston *et al*. [4] is the idea that distinct groups can be identified within the wider Wairau Bar cemetery population prior to isotopic analysis. Kinaston *et al*. argue that certain cultural markers differentiate Burials 1–7 from the rest of the cemetery population ([4]: 2). Their goal is to assess whether the supposed cultural differences are apparent in the isotopic signatures or if the cemetery represents a homogenous isotopic group. While we recognise the obvious utility of defining sub-groups in such an analysis, we have identified two concerns with their construction and application in this case. Below we consider each of these in turn.

### Group construction

The basis of sub-dividing the cemetery population is ostensibly a distinction between Burials 1–7 (Group 1) and the remaining Burials 8–44 (Group 2/3) due to differences in burial position, burial wealth and spatial location. However, in practice both burial wealth and burial position are used only to emphasise the distinction between groups rather than as exclusive criteria for membership. Reference to ‘burial position’ in S1 Table makes this clear. Here it can be seen that whilst the majority of Group 1 individuals were interred in the prone position, two individuals were not, and at least six individuals in Group 2/3 were also interred in the prone position. Likewise, while the authors note that the average amount of grave goods is much higher in Group 1, some burials with many grave goods are included in Group 2/3. Moreover, it is not certain that the burial position and wealth data are reliable indicators of difference since the Group 2/3 burials are known to have been much more disturbed and fragmented at recovery than the relatively intact Group 1 [8]. Consequently some of the perceived variation may be due to taphonomic bias.

Effectively this means that Kinaston *et al*. [4] define groups primarily by their spatial location, with the other attributes being used as suggestive secondary evidence indicating that the spatial differences might be culturally meaningful.

**Fig 1:**
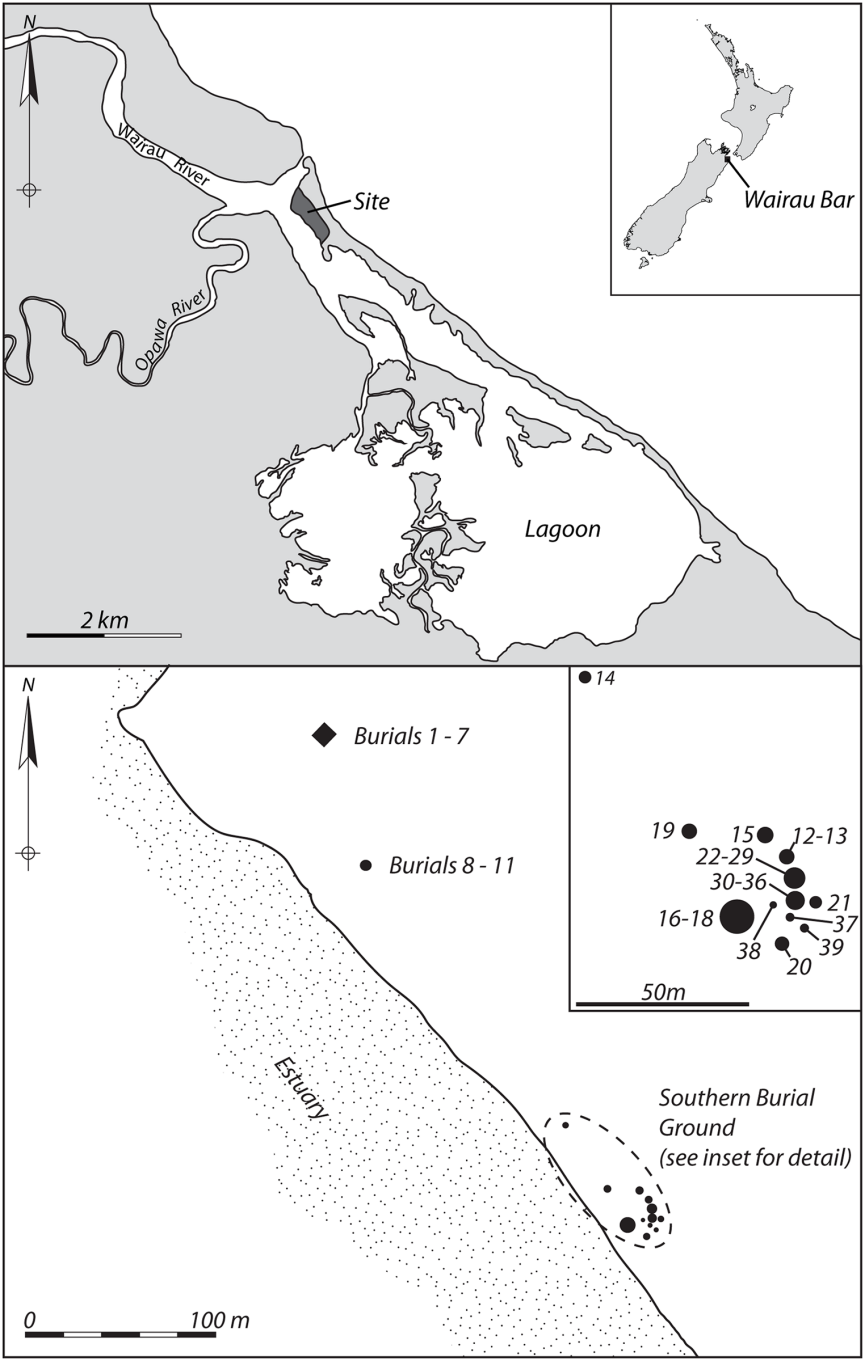
Location of the Wairau Bar site and the burial groups. **Map and** burial location information adapted from Brooks *et al*. 2011).

The use of a single defining criterion for a group in concert with supporting information is, arguably, valid. The spatial clustering of burials in mortuary contexts has been demonstrated to have social correlates such as kinship groups [9, 10] but the veracity of these must be statistically robust, and, moreover, independent of the subsequent explanatory framework and variables tested. In this instance we believe that the spatial criteria for developing analytical groups adopted by Kinaston *et al*. is inconsistent. Duff [11] argues for the existence of three burial groups at the site: Burials 1–7 (Group 1), Burials 8–11 (Group 2) and a ‘southern burial ground’ comprising Burials 12–44 (Group 3), which extends over roughly one-hundred metres (fifty metres excluding burial 14) along the edge of the lagoon and contains discrete burial clusters of comparable size to Groups 1 and 2 [12] (Fig 1). These designations are adopted by the authors; however, for their purposes, they combine Burials 8–11 and 12–44 into a single group labelled Group 2/3, despite the Group 2 burials being spatially closer to Group 1.

The effect of this decision is that Group 1 is defined using different spatial criteria from Group 2/3. Group 1 consists of a small, coherent group of well-preserved individuals aligned with each other in 9 x 6 m unit, while Group 2/3 (as evidenced by its name) is a large composite or ‘leftovers’ group from multiple units spread over several hundred metres of site. This is important to take into consideration when interpreting the results of statistical analysis comparing these groups.

### Statistical analysis of dietary stable isotopes

Kinaston *et al*. carry out a series of statistical tests to ascertain if Group 1 and Group 2/3 are dietarily distinct. In the first instance their analysis involves a comparison of the Group 1 and Group 2/3 mean δ^13^C and δ^15^N values. The authors report no statistically significant difference in δ^13^C values; however, the Group 1 average δ^15^N value, representing the plant/protein portion of diet was significantly lower (Student’s t-test p = 0.025) than Group 2/3. This result comes with the caveat that a significant result was not found after adjusting for sex (p = 0.150); therefore, caution must be used when interpreting the initial significant result. This is clearly a reflection of the influence a predominance of males in Group 1 (n = 5 of six individuals) has on the unadjusted value. At this point then, no reliable significant difference has been found between these groups to suggest that they are distinct populations.

In the second instance, a significant difference in the variance of δ^13^C values (but not δ^15^N values) between groups (Levene’s test p = 0.014) is invoked as more evidence for dietary difference or change [4]. We argue that caution should also be exercised when interpreting the variance data, particularly given the lack of difference in mean δ^13^C values. Drawing upon our earlier discussion of group construction, it is clear that Group 1 and Group 2/3 are not equivalent, with the latter consisting of all burials across the site not assigned to Group 1. Under such conditions we should expect Group 2/3 to be more variable than Group 1 as an artefact of classification. That a discrete cluster of burials exhibits less dietary variability than the site as a whole is unsurprising and currently the only reliable inference we can make is that the Group 1 burials exhibit a subset of the range of values exhibited by the population of the site as a whole.

Kinaston *et al*.*’s* final statistical analysis of dietary isotopes combined the δ^15^N and δ^13^C values with the ^87^Sr/^86^Sr ratios in a principal component analysis. They note:

*“Two principal components were sufficient to explain 58% and 35% respectively (total 92%) of the variance in the data*. *Component one reflected high carbon and nitrogen values while component two reflected high strontium values with a moderate negative carbon loading…Individuals from Group 1 are clearly separable from those in Group 2/3 (even using only the second component representing mainly strontium values) and one individual from Group 2 is at the edge of this cluster”* ([4]: 8).

While Group 1 does appear distinct in the PCA plot, a careful examination of the components shows a lack of support for distinction of groups based on diet alone. Component 1, reflecting high carbon and nitrogen (i.e. dietary) values, does not separate Group 1. Instead, the Group 1 individuals fit well within the range of values present in Group 2/3. Kinaston *et al*. ([4]: 9) remark that the groups are clearly separable “even using only the second component representing mainly strontium values”. In fact the second component is probably the sole driver of separation between the groups, even though it accounts for less variance. The separation evident in the PCA simply reflects a difference in ^87^Sr/^86^Sr values (*i*.*e*. a proxy for place of childhood residence) and does not lend further support to the presence of dietary distinctions between groups.

## Results: Alternative Statistical Analysis of Dietary Stable Isotopes

We suggest that greater statistical certainty can be achieved by maintaining the independence of new data sets from existing classifications during initial analysis. Cultural or biological factors, such as burial location or sex, need not be considered predictors of isotopic variation. Instead, stable isotope data may exhibit independent patterns. Considering this, we begin our analysis by searching for patterns based solely on the δ^15^N and δ^13^C values. We carry out a cluster analysis, which we then cross-check against cultural and biological variables to determine the most likely factor(s) behind any patterns.

### Hierarchical cluster analysis

**Fig 2:**
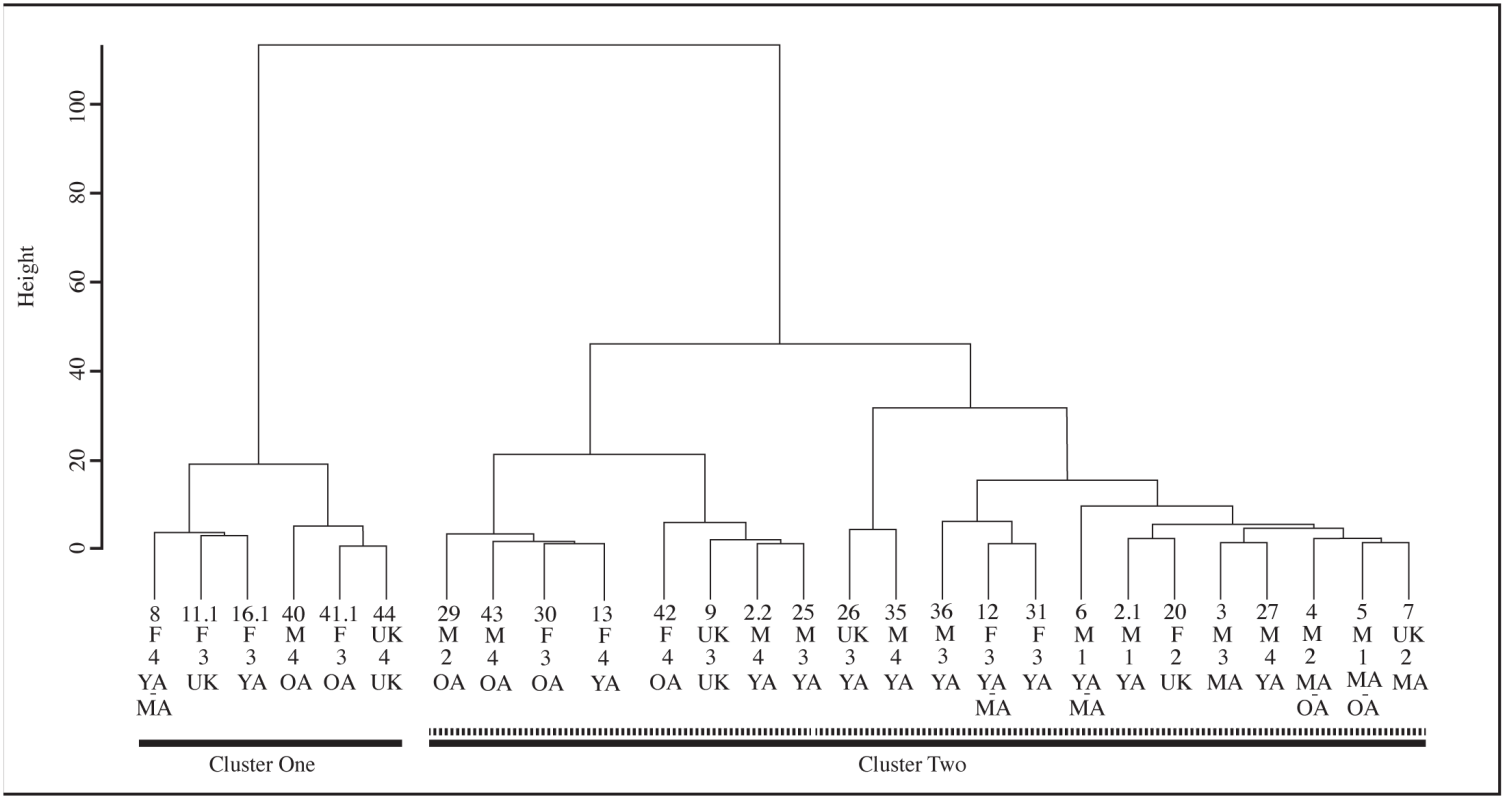
Ward's cluster analysis of dietary stable isotope (δ^15^N and δ^13^C) values from Wairau Bar with associated age, sex and wealth information.

**Fig 3:**
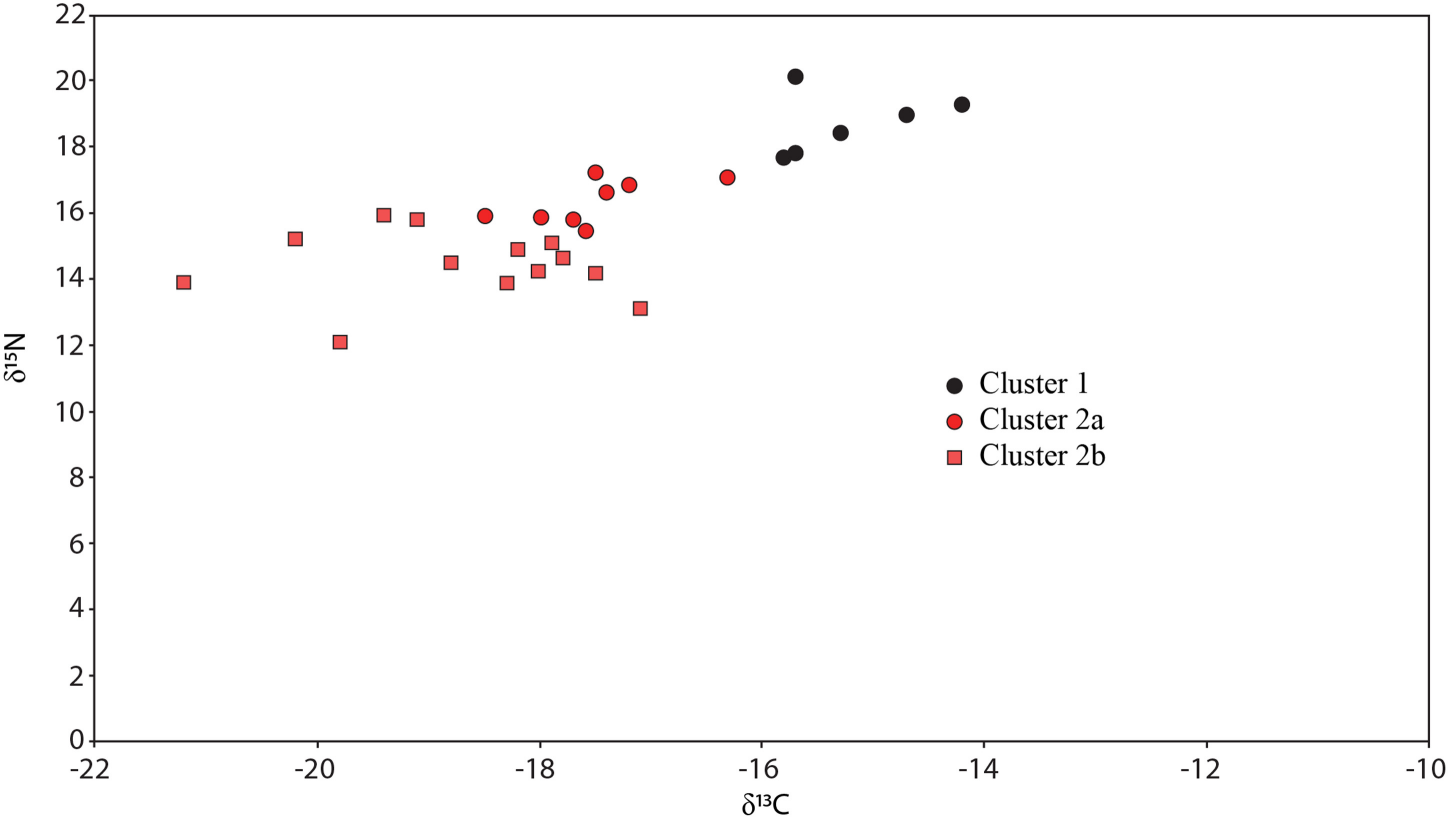
Scatter plot of the Clusters determined by Ward's Linkage hierarchical clustering.

Using the δ^15^N and δ^13^C values we carried out a hierarchical agglomerative cluster analysis using Ward’s linkage [13]. The resulting dendogram (Fig 2) presents two strong clusters. The first, (Fig 2; Cluster One) is a clear group of six burials with high δ^15^N and δ^13^C values. The second cluster contains the remaining 21 burials and has two weaker, second-order sub-groups. While we will consider these sub-groups separately in the following section, overlap of the δ^15^N and/or δ^13^C values is present (Fig 3). Interpretation of these sub-groups as distinct is, therefore, made with caution.

The most important observation from this cluster analysis is that the culturally defined groups used by Kinaston *et al*. [4] are not replicated when only the dietary isotopes are considered. Rather, all of Group 1 (Burials 1–7) is mixed with burials from Group 2/3 in the second, large cluster. Performing a t-test revealed no significant differences (δ^13^C p = 0.31; δ^15^N p = 0.10) between Group 1 and Group 2/3 within Cluster Two even without correcting for sex.

We consider it important that most (n = 4 of 6) of Cluster One are female (Fig 3). This group falls outside the local rat and dog ranges reported by Kinaston *et al*. ([4]: their Fig 4) and is much closer to marine mammal values, perhaps hinting at an increased marine dietary focus in this group. A t-test of the burial population with confirmed sex estimates found significant difference in the mean δ^15^N value of the sexes (p = 0.02) but not δ^13^C (p = 0.114). A similar result is noted by Kinaston *et al*. [14] for the Teouma site in Vanuatu. At Teouma δ^15^N and not δ^13^C were found to be significantly higher in males. The authors consider two possible explanations: that this trend suggests sexual division of labour with respect to the procurement of particular food types, consistent with similar practices observed in modern day Pacific Islands communities, or that food consumption was moderated along sexual lines with males enjoying greater access to higher prestige foods due to higher social status ([14]: 14). Teouma is an interesting parallel to Wairau Bar since both sites were occupied during an early phase of colonisation. The observation of sex-based differences in food consumption due to either division of labour or differential status provides interesting insight into the social structure of early period sites.

**Fig 4:**
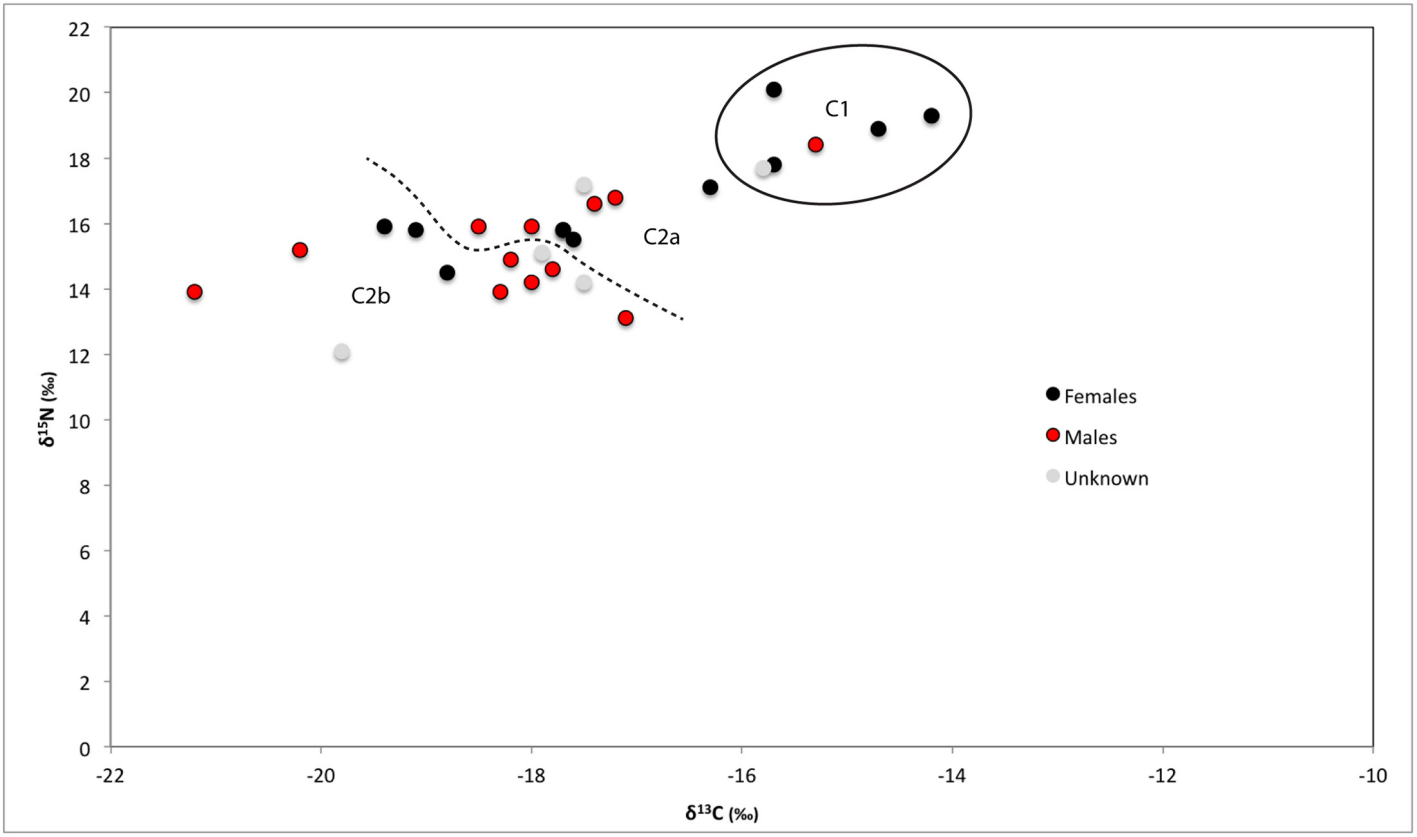
Human bone collagen δ^15^N and δ^13^C values from Wairau Bar coded with the sex of the individual.

**Fig 5:**
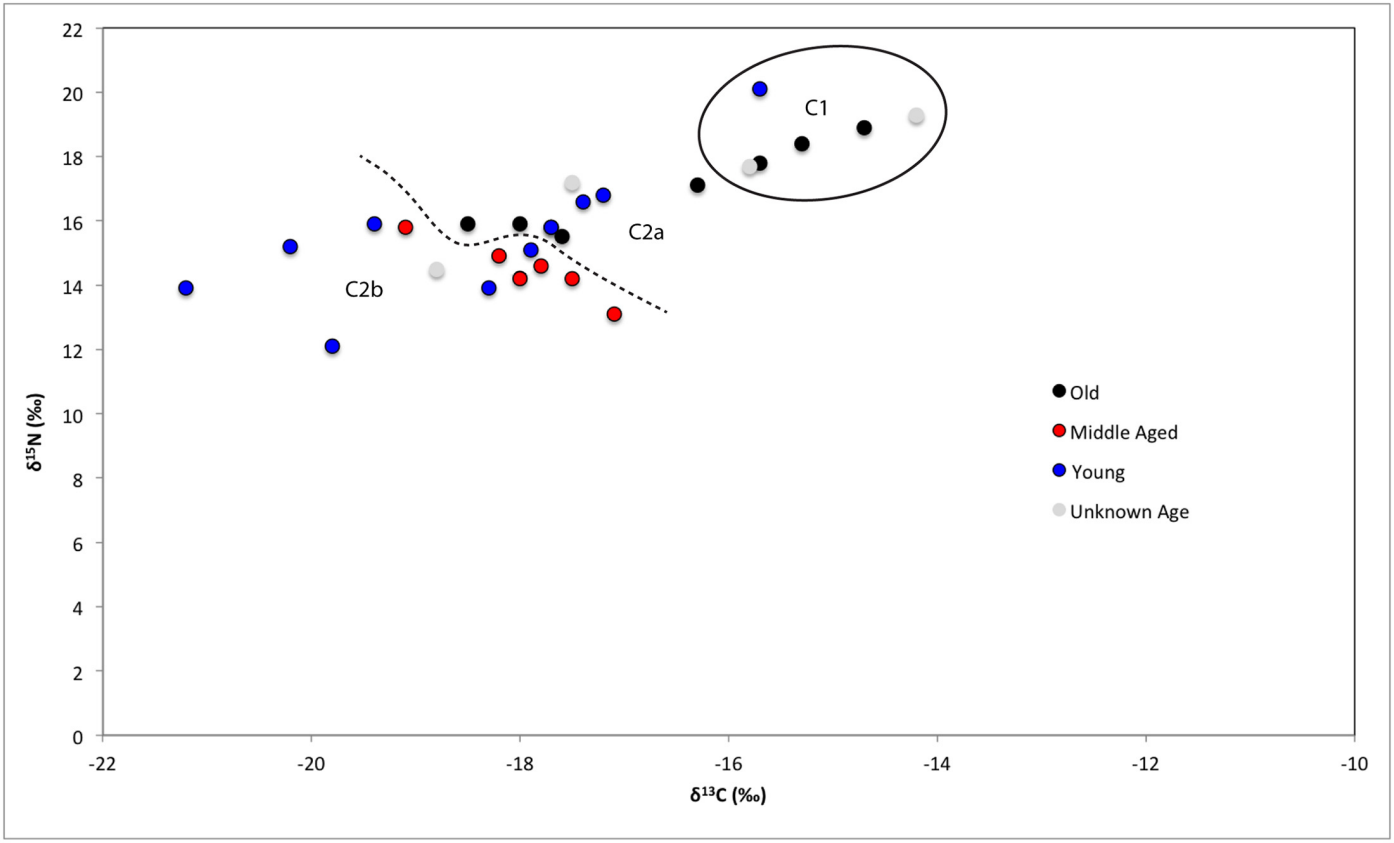
Human bone collagen δ^15^N and δ^13^C values from Wairau Bar coded with information about individual age at death.

**Fig 6:**
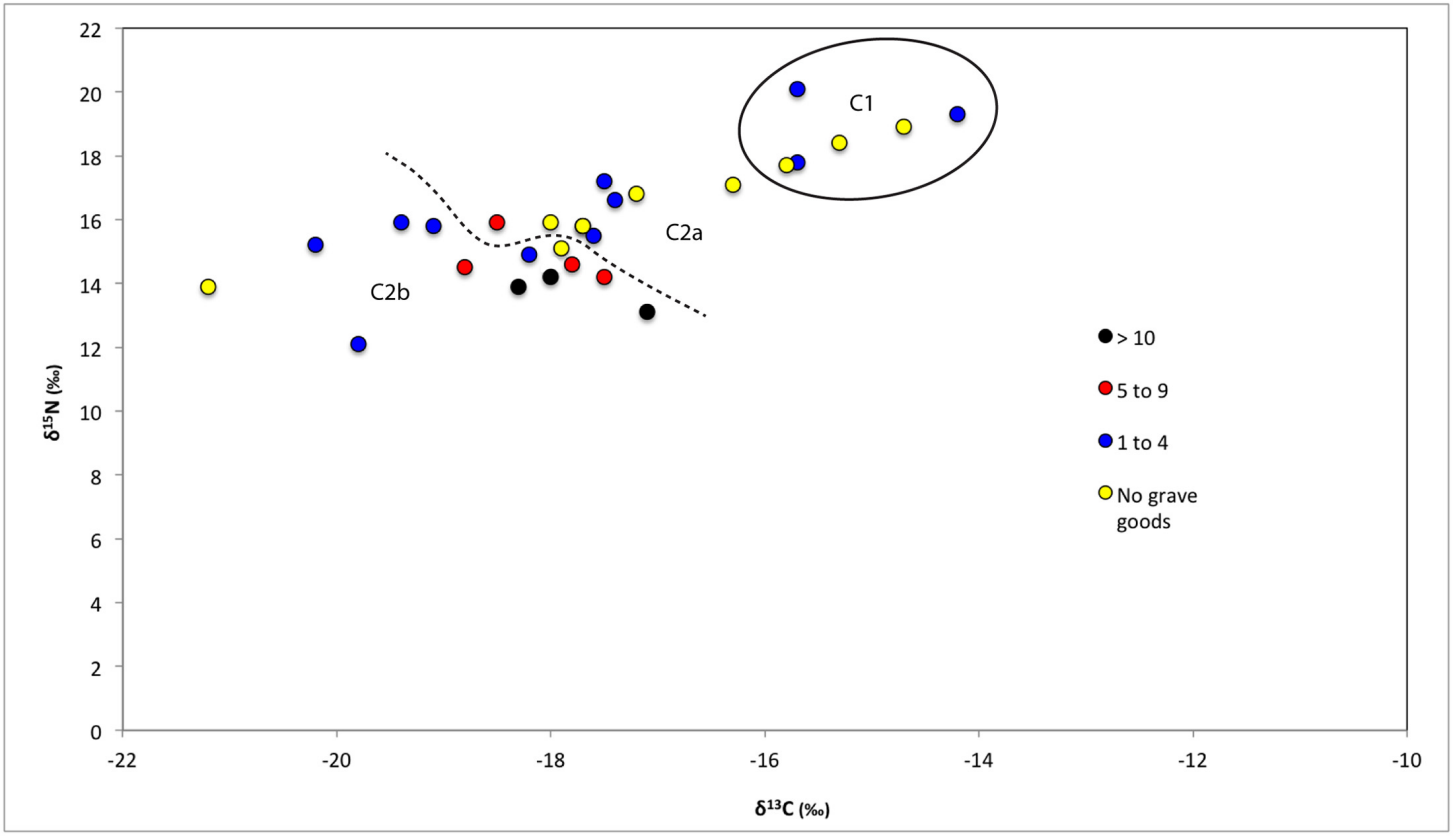
Human bone collagen δ^15^N and δ^13^C values from Wairau Bar coded with grave goods information.

**Fig 7:**
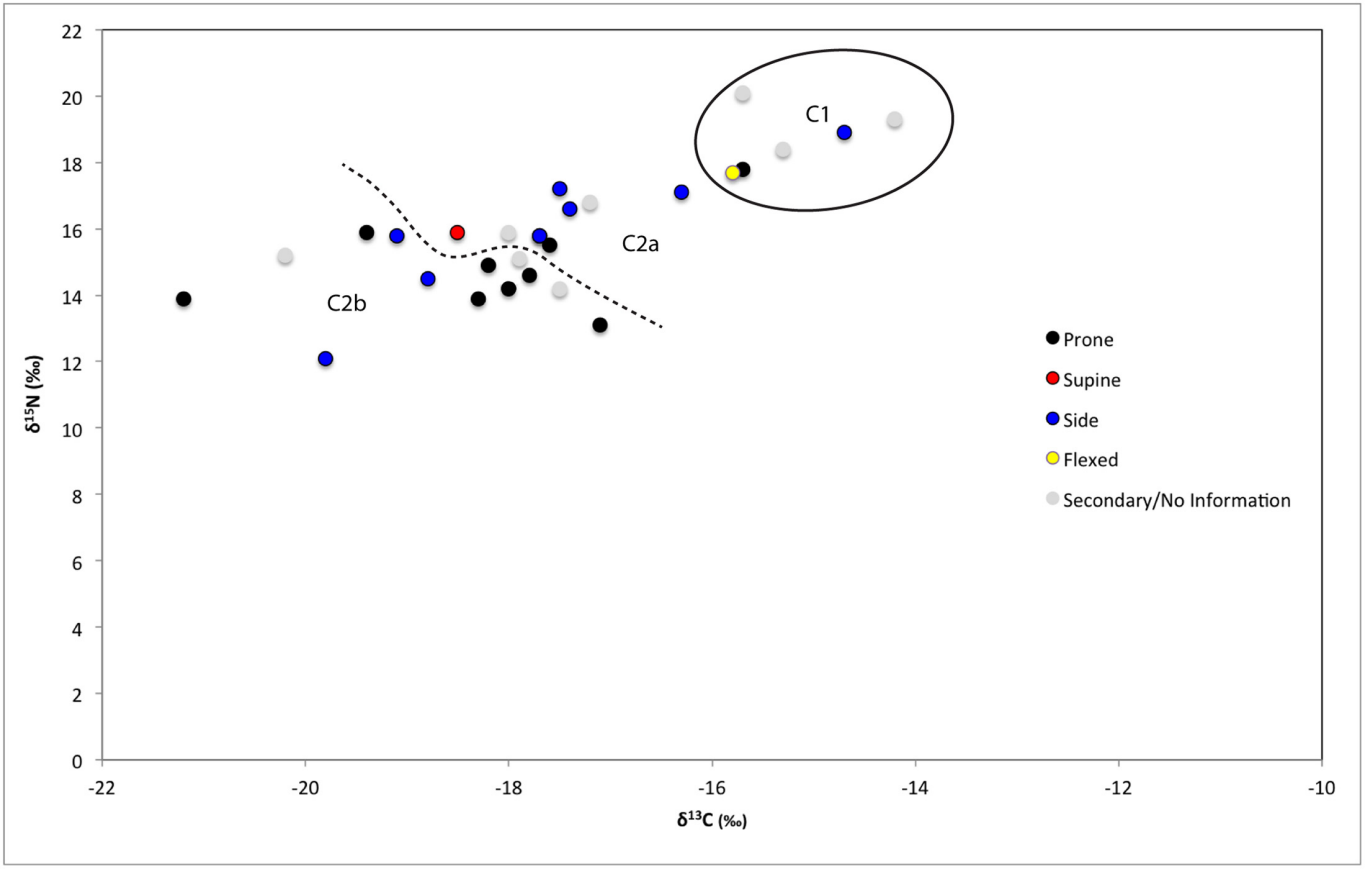
Human bone collagen δ^15^N and δ^13^C values from Wairau Bar coded with burial position information.

Despite the statistical support for sex-based differences it is also true that there is a large degree of overlap between females and males (Fig 4). Just over half of the females fall comfortably within the range occupied by most males. Indeed a t-test confirms there are no significant differences between males and females in Cluster Two (δ^13^C p = 0.6; δ^15^N p = 0.073). This suggests that the Cluster One individuals present a distinct group of mostly female outliers from the population as a whole. Visual assessment of Figs 4–7 suggests that Cluster One may also be characterised by older individuals with fewer grave goods. However, statistical analysis revealed no significance in these patterns.

### Model selection methods

Our analysis (above) of the δ^15^N and δ^13^C data suggests that other factors such as sex may have more explanatory power than the burial groupings employed by Kinaston *et al*. [4]. In order to assess competing explanations for the patterns exhibited in the isotope data we carried out a model selection of multivariate regression models using the Akaike Information Criterion (AIC) and Bayesian Information Criterion (BIC). Beginning with Kinaston Groups, Age, Sex and Grave Goods (Models A and D) and Duff Groups, Age, Sex and Grave Goods (Models B and C), a stepwise selection of the best explanatory combination of covariates was carried out (see [15, 16]). During this process AIC punishes model complexity less than BIC [16] and is therefore less likely to produce an overly simplistic model. Because of this relative conservatism we report AIC results in this paper, but also include the results of BIC model selection (S2 Table). The BIC analysis produced the same patterns as the AIC. Burial position was removed from our analysis because of possible bias in the data caused by taphonomy (see above).

Table 1 shows the results of the model selection using AIC, where the best quality model is represented by low AIC value and high weight. For δ^15^N, the best model involves the single covariate sex (Model A [Kinaston Groups], Model B [Duff Groups], Model C [Groups absent]). This finding agrees with the significant difference in mean δ^15^N of males and females reported above, and the fact that Kinaston *et al*. [4] found no significant difference between their groups after controlling for sex. For δ^13^C, analysis of Models D and E finds that a combination of grave goods and burial group (either Duff or Kinaston) are the best models. Given that we found no significant difference in mean δ^13^C values between the sexes, and Kinaston *et al*. [4] report no significant difference in mean δ^13^C values between their groups, this finding probably reflects difference in the variance data. But, as we argue above, the fact that Kinaston et al’s Group 2/3 has more variation in δ^13^C values than Group 1 is likely to be a product of classification. Consequently we must question the validity of this result. Removing burial group and testing only the objectively derived variables (Model E), we see that the importance of grave goods is reduced and variation in δ^13^C is again best explained by the single covariate sex.

**Table 1:** Model selection using Akaike Information Criterion (AIC).

Response Variable	Model Steps	Covariates	AIC	Weights
δ^15^N (‰)	**Model A**			
	**1**	Kinaston Group, Age, Sex, Grave Goods	26.35	0.046
	**2**	Kinaston Group, Age, Sex	24.5	0.115
	**3**	Age, Sex	22.6	0.297
	**4**	Sex	21.4	0.541
δ^15^N (‰)	**Model B**			
	**1**	Duff Group, Age, Sex, Grave Goods	26.49	0.043
	**2**	Duff Group, Age, Sex	24.56	0.113
	**3**	Age, Sex	22.6	0.301
	**4**	Sex	21.4	0.541
	**Model C**			
	**1**	Age, Sex, Grave Goods	24.58	0.117
	**2**	Age, Sex	22.6	0.315
	**3**	Sex	21.43	0.566
δ^13^C (‰)	**Model D**			
	**1**	Kinaston Group, Age, Sex, Grave Goods	27.4	0.092
	**2**	Kinaston Group, Sex, Grave Goods	25.43	0.247
	**3**	Kinaston Group, Grave Goods	23.46	0.661
δ^13^C (‰)	**Model E**			
	**1**	Duff Group, Age, Sex, Grave Goods	28.2	0.133
	**2**	Duff Group, Sex, Grave Goods	26.2	0.362
	**3**	Duff Group, Grave Goods	25.54	0.504
δ^13^C (‰)	**Model F**			
	**1**	Age, Sex, Grave Goods	28.63	0.112
	**2**	Age, Sex	26.7	0.293
	***3***	Sex	25.28	0.596

## Strontium Isotopes and Population Mobility

Kinaston *et al*. argue for clear separation of Group 1 from Group 2/3 according to ^87^Sr/^86^Sr ratios. In particular, Kinaston *et al*. ([4]:7) state that:

“…*given that the strontium results from Group 2/3 are closer to those of the local dog sample than to Group 1*, *a reasonable interpretation of this pattern is that the Group 1 individuals were immigrants to the site while some of the Group 2/3 individuals had resided in or near the Wairau Bar region during childhood”*.

While it is true that Group 2/3 have, on average, higher values than Group 1, this interpretation is not consistent with the authors’ advice that:

*“variations in human strontium isotope values greater than two standard deviations from the mean of the local range are then considered to be most likely ‘non-local’ to the site”* ([4]: 4).

**Fig 8:**
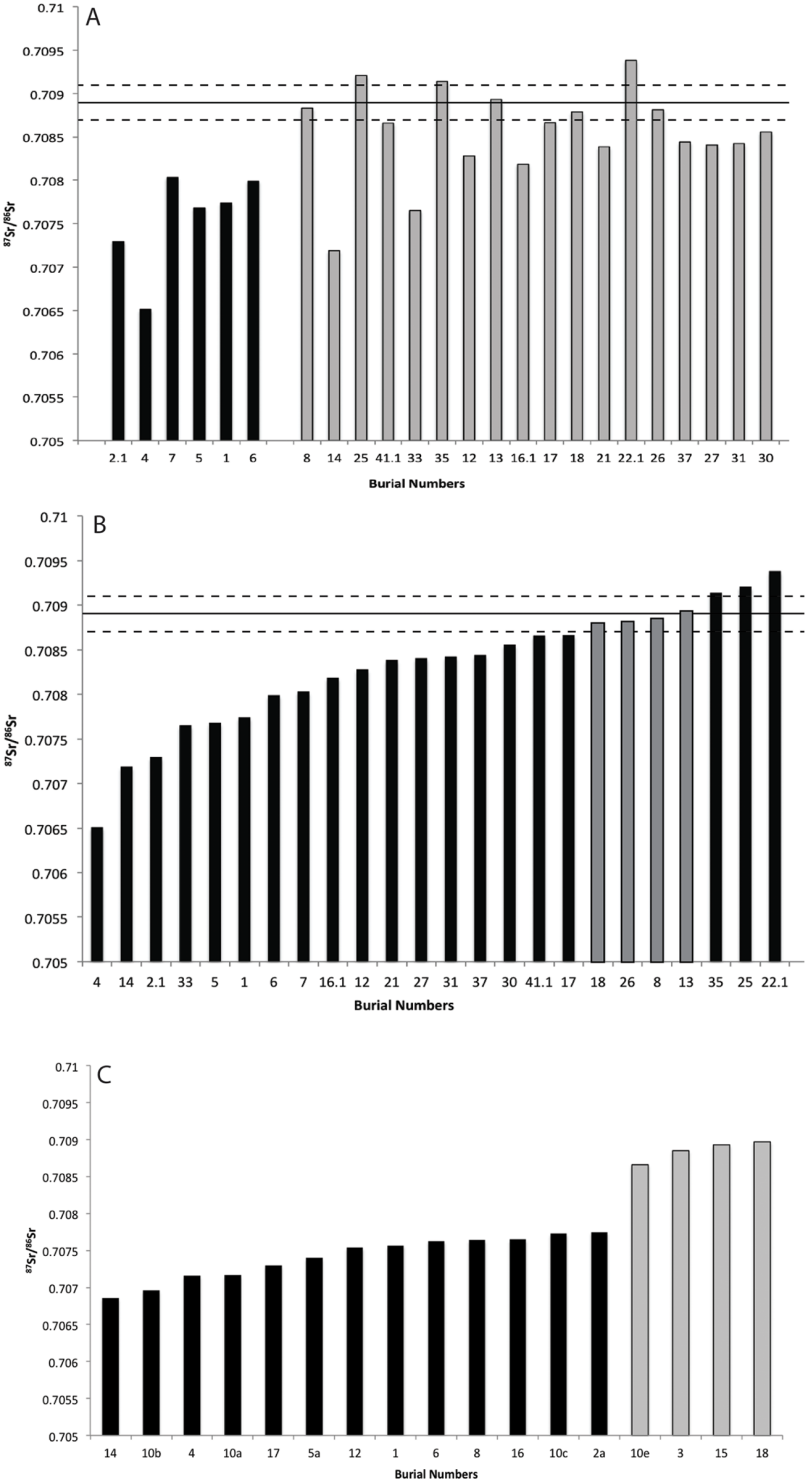
Strontium isotope data from Pacific Island samples. A—Reproduction of Kinaston et al. (2013, Fig 5) showing the strontium values of individuals within their Group 1 (black) and Group 2/3 (grey). B. Strontium values from Wairau Bar individuals organised into rank order, grey bars represent individuals within two standard deviations of the mean dog value. C—Strontium isotope data from Teouma, Vanuatu (Bentley et al. 2007). Grey bars represent individuals considered to be outliers or immigrants to the site.

Based on the authors figure (reproduced here in Fig 8a), only four individuals fall within two standard deviations of the mean strontium values from dog samples, which are taken as the proxy for a local signature. Therefore, while it is correct to say that some of the Group 2/3 individuals may be local, it would be more accurate according to their standard, to argue that we can be 95% sure that 20 individuals are non-local.

Re-organising the data into rank order is revealing (Fig 8b). The resulting distribution shows that the ^87^Sr/^86^Sr ratios may actually form a far more continuous group than Kinaston *et al*. suggest. Moreover it is evident that some Group 2/3 burials (e.g. burials 14 and 33) have very low values similar to the allegedly immigrant Group 1 (a fact discussed by the authors). Overall there is not an extreme difference between individuals assigned to Group 1 and members of Group 2/3 despite differences in their average values, and there are no clear groups of outliers. This contrasts with results from Teouma, another Pacific Island colonisation phase site where the presence of immigrants has been argued. Fig 8c presents the ^87^Sr/^86^Sr results presented by Bentley *et al*. [17]. Bentley *et al*. argue that the four individuals on the right of the graph are outliers from the main population, a likely indication of them being immigrants to the site. The stepped appearance of the graph presents a strong contrast to the continuous slope observed at Wairau Bar. We accept that geological variation coupled with the current lack of an adequate strontium baseline in New Zealand makes the detection of differences as distinct as those at Teouma unlikely; nevertheless, the comparison is a cautionary note for the assignment of Burials 1–7 as a discrete group of immigrants.

## Discussion and Implications

As the so-called ‘type site’ of the New Zealand Archaic, Wairau Bar has an important explanatory role in New Zealand archaeology. As well as providing evidence for a range of activities that indicate early economic behaviour and settlement strategy [18], the site provides the largest collection of well-provenanced, early *koiwi tangata* (human remains) in New Zealand. Thus, it offers a unique opportunity to apply biological anthropology methods to the study of New Zealand’s earliest human population. Until recently the analysis of skeletal remains from the site has focussed on the physical characteristics, lifestyles and pathologies of individuals [8, 19]. Variation within the cemetery population has also been considered, with sex, relative wealth and/or status, and temporal change being invoked to account for differences [10, 20, 21]. An early component of these analyses was the distinction drawn between Burial Group 1 and the remaining burials based primarily on burial location within the site and grave goods. Kinaston *et al*. [4] use this distinction as a starting point for their analysis of stable isotopes, searching for differences between Group 1 and the rest of the cemetery. Doing so, we argue, gives too much weight to the spatial location of burials, and less to factors such as the age or sex of the individuals. As an alternative, we have treated the stable isotope data independently and have sought to test a broad range of models to come up with the best explanation of variation.

The alternative analysis conducted in this paper suggests two important things about diet: (1) that sex is the best explanatory variable for dietary variation and (2) that there is little evidence of dietary distinction based on burial location at Wairau Bar. Our analysis showed the most dietarily-distinct individuals to be those with high δ^15^N and δ^13^C values consistent with a diet containing a higher marine component. These individuals formed a cluster of predominantly female burials with relatively few grave goods.

In the first instance this pattern could be indicative of gendered differences in food procurement activities. Drawing upon a subset of data from the Standard Cross Cultural Sample (SCCS), Marlowe [22] showed that, within foraging societies, females target reliable foods while males tend towards energy rich, less reliable food sources. Sexual division of labour during food procurement is a consistent pattern throughout Polynesia, although its implementation varies [23]. In late period Maori society Firth [24] notes gendered subsistence practices that are generally consistent with Marlowe’s [22] findings: men tended to carry out expeditions for less reliable food sources, while females targeted more abundant, reliable foods closer to the domestic base. For example, and in common with most Pacific societies, inshore fishing and shellfish gathering were preferentially performed by women and accounted for a larger proportion of the diet than the rare, risky and less productive offshore fishing activities of men.

Many models of early New Zealand settlement suggest groups actively exploited a wide territory via a network of coastal village bases and supplementary restricted function sites [25, 26]. If food procurement amongst early Maori had gender divisions comparable to those of the later period, it is possible that females and males accumulated different proportions of marine and terrestrial foods in their diet. In the case of Wairau Bar, the vast estuarine setting would have provided a range of reliable subsistence resources to exploit. Conversely, hunting activities may have rapidly depleted local large-bodied game [27] fuelling range expansion, possibly seen in the establishment of specialist camps within a few days journey of the site [25]. This model may account for sex-based variation in diet simply due to the proportion of time spent food gathering in different environmental zones. Although the products of food gathering were probably pooled, habitual sustenance during food procurement forays in different environments might in itself be enough to skew isotopic ratios.

An alternative hypothesis indicated by Figs 5 and 6, and our AIC analysis of the δ^13^C data is that grave goods may have some spatial patterning in the site (see also [11, 20]) and this may also predict dietary variation. Although contentious [28], if we accept that abundance of mortuary offerings represents a proxy of the wealth/status of individuals in life (e.g. [29]), then the Cluster One dietary outliers appear to have been relatively low wealth or status. Historical evidence attests to the existence of status related dietary divisions in Polynesia [24, 30, 31]. Principally, oily, fatty meats were considered of higher status [32, 33], although more mundane foods could gain value through labour-intensive preparation methods [33]. It is possible that meats like moa, which conform to a generic description of a food with high status in Polynesia, were less available to individuals such as those in Cluster One due to their social position. However we were not able to demonstrate this association with robust statistical significance, and problems with the existing unsystematic spatial categorisation of burials inhibits our ability to interpret the AIC findings. This matter warrants more research.

Our reassessment of the strontium isotope data also challenges aspects of the model proposed by Kinaston *et al*. [4]. While we accept that Burials 1–7 fall in the lower end of the range of ^87^Sr/^86^Sr ratios, we argue that the shape of the data across the site suggests they are part of a continuum rather than a distinct group (e.g. Fig 8). The range of values expressed in the sample is very large, and if we strictly follow the guideline that local individuals are those who have ^87^Sr/^86^Sr ratios within two standard deviations of the mean value for local dogs then the majority of individuals (20 of 24) interred at Wairau Bar are non-locals. This data, however, is difficult to interpret without better baseline information on regional variation in biologically available strontium. The range of geological strontium ratios reported by Kinaston *et al*. [4]for the Wairau Valley is so large that it easily encompasses all of the variation in the archaeological sample. The ratios reported for Pacific Islands [4] are comparatively narrow in range, but remarkable for being considerably lower than the archaeological samples at Wairau Bar. A complicating factor is that coastal and island populations have repeatedly been shown to have ^87^Sr/^86^Sr ratios approaching that of seawater (0.7092) due to the influence of a marine based diet, and this can elevate ratios above that of the local geology [17]. In other words, whilst we agree with Kinaston *et al*. that it is likely the large variation in ^87^Sr/^86^Sr ratios exhibited by the Wairau Bar sample is indicative of a very mobile population, we believe we do not yet know enough to make any secure claims about origins.

## Conclusion

We agree with Kinaston *et al*. that Wairau Bar appears to be a special site in the colonising phase of New Zealand and that, particularly on the basis of material culture, there are differences between individuals in the site. However, here we have argued that the stable isotope data does not support the identification of spatially coherent clusters of individuals with diets different from the majority of the population at the site. Instead, we find that dietary differences were most probably influenced by the sex of individuals and that there is no clear isotopic evidence to support the designation of a group of Wairau Bar individuals as the first immigrants. We would argue that these results tell us something potentially more interesting about the social dimensions of diet and mobility in early New Zealand.

At a broad level this study has shown the potential benefits of multi-factorial analyses of stable isotope data in the Pacific Islands. Traditional single hypothesis-testing approaches often employ groups that increase the potential of finding positive results, thereby introducing a degree of circularity and decreasing the likelihood of further tests being carried out. As large-scale samples like Wairau Bar are relatively rare in Pacific Island archaeology, we advocate a broad exploration of variables that ensures the maximum amount of information is returned. The development of better strontium baselines would aid in future analysis of mobility, as may the collection of δ^18^O data, which, given the variation in precipitation and temperature in New Zealand is likely to be informative.

## Supporting Information

S1 Table Demographic, burial, wealth and isotopic data from the human remains at Wairau Bar (adapted from Kinaston et al. 2013: S2 Table). (DOCX)

S2 Table The results of stepwise model selection using both AIC and BIC criterions. (DOCX)
