# Supplementary material for: The First New Zealanders? An Alternative Interpretation of Stable Isotope Data from Wairau Bar, New Zealand
Source: PLoS One. 2015 Oct 28;10(10):e0135214. doi: 10.1371/journal.pone.0135214 (PMC4624984; doi:10.1371/journal.pone.0135214)
Supplement: S1 Table — (DOCX) [file pone.0135214.s001.docx]

S1 Table. Demographic, burial, wealth and isotopic data from the human remains at Wairau Bar (adapted from Kinaston et al. 2013: S2 Table).

| Burial | Duff Group | Kinaston Group | Age^a^ | Sex^b^ | Position^c^ | Wealth^d^ | ^87^Sr/^86^Sr | ±^87^Sr/^86^Sr (2 S.E.) | %N | δ^15^N (‰) | %C | δ^13^C (‰) | C:N |
| --- | --- | --- | --- | --- | --- | --- | --- | --- | --- | --- | --- | --- | --- |
|  |  |  |  |  |  |  |  |  |  |  |  |  |  |
| 1 | 1 | 1 | YA-MA | F | Prone | No info | 0.707741 | 0.000014 |  |  |  |  |  |
| 2.1 | 1 | 1 | YA | M | Prone | 1 | 0.707297 | 0.000012 | 9.9 | 13.9 | 29.3 | -18.3 | 3.4 |
| 2.2 | 1 | 1 | YA | M? | No info | 4 |  |  | 9.1 | 16.8 | 27.9 | -17.2 | 3.6 |
| 3 | 1 | 1 | MA | M | Prone | 3 |  |  | 9.1 | 14.9 | 27.6 | -18.2 | 3.5 |
| 4 | 1 | 1 | MA-OA | M | Prone | 2 | 0.70651 | 0.000009 | 8.7 | 14.6 | 26.2 | -17.8 | 3.5 |
| 5 | 1 | 1 | MA-OA | M? | Prone | 1 | 0.70768 | 0.000014 | 12.2 | 14.2 | 36.4 | -18 | 3.5 |
| 6 | 1 | 1 | YA-MA | M | Prone | 1 | 0.70799 | 0.000015 | 8.9 | 13.1 | 22.6 | -17.1 | 3 |
| 7 | 1 | 1 | MA | UK | Secondary | 2 | 0.708032 | 0.000014 | 14.3 | 14.2 | 42.5 | -17.5 | 3.5 |
| 8 | 2 | 2 & 3 | OA | F | Left side | 4 | 0.708832 | 0.000012 | 13.4 | 18.9 | 39.2 | -14.7 | 3.4 |
| 9 | 2 | 2 & 3 | UK | UK | Right side | 3 |  |  | 13 | 17.2 | 40.3 | -17.5 | 3.6 |
| 11.1 | 2 | 2 & 3 | UK | F | No info | 3 |  |  | 12.9 | 19.3 | 38 | -14.2 | 3.4 |
| 12 | 3 | 2 & 3 | YA-MA | F | Right side | 3 | 0.708279 | 0.000015 | 14.2 | 15.8 | 41.4 | -19.1 | 3.4 |
| 13 | 3 | 2 & 3 | YA | F | Right side | 4 | 0.708931 | 0.000012 | 17.7 | 15.8 | 52.7 | -17.7 | 3.5 |
| 14 | 3 | 2 & 3 | OA | M? | Prone | 2 | 0.707188 | 0.000012 |  |  |  |  |  |
| *15* | *3* | 2 & 3 | *OA* | *F?* | *Flexed* | *3* |  |  | *7.9* | *14.5* | *25* | *-18* | *3.7* |
| 16.1 | 3 | 2 & 3 | YA | F | Secondary | 3 | 0.708185 | 0.000013 | 10.5 | 20.1 | 32.1 | -15.7 | 3.6 |
| *16.2* | *3* | 2 & 3 | *OA* | *F* | *Commingled* | *3* |  |  | *8* | *17.6* | *25.7* | *-16.4* | *3.8* |
| *17* | *3* | 2 & 3 | *YA* | *F* | *Supine* | *4* | *0.708664* | *0.000012* | *11.4* | *15.8* | *41.1* | *-17.7* | *4.2* |
| *18* | *3* | 2 & 3 | *YA-MA* | *F* | *Secondary* | *4* | *0.708787* | *0.00001* | *12.6* | *19.3* | *43.1* | *-15.9* | *4* |
| *19* | *3* | 2 & 3 | *YA* | *M?* | *Prone* | *4* |  |  | *10.3* | *13.9* | *33.9* | *-19.8* | *3.8* |
| 20 | 3 | 2 & 3 | UK | F? | Right side | 2 |  |  | 9.5 | 14.5 | 29.2 | -18.8 | 3.6 |
| 21 | 3 | 2 & 3 | MA | F | Supine | 4 | 0.708386 | 0.000011 |  |  |  |  |  |
| 22.1 | 3 | 2 & 3 | YA | F | Supine | 3 | 0.709382 | 0.00001 |  |  |  |  |  |
| *22.2* | *3* | 2 & 3 | *OA* | *F* | *Commingled* | *3* |  |  | *5* | *17.2* | *17.8* | *-18* | *4.2* |
| *24* | *3* | 2 & 3 | *OA?* | *M* | *Supine* | *4* |  |  | *3.5* | *14.1* | *12.1* | *-17.4* | *4* |
| 25 | 3 | 2 & 3 | YA | M | Left side | 3 | 0.709208 | 0.000013 | 11 | 16.6 | 33.4 | -17.4 | 3.6 |
| 26 | 3 | 2 & 3 | YA | UK | Left side | 3 | 0.708812 | 0.000011 | 16.3 | 12.1 | 48 | -19.8 | 3.4 |
| 27 | 3 | 2 & 3 | YA | UK | No info | 4 | 0.708406 | 0.000013 | 15.9 | 15.1 | 47.6 | -17.9 | 3.5 |
| *28* | *3* | 2 & 3 | *UK* | *F* | *Crouched* | *3* |  |  | *8* | *15.9* | *25.2* | *-18.8* | *3.7* |
| 29 | 3 | 2 & 3 | OA | M | Supine | 2 |  |  | 12.8 | 15.9 | 36.1 | -18.5 | 3.3 |
| 30 | 3 | 2 & 3 | OA | F | Prone | 3 | 0.708557 | 0.000013 | 10.7 | 15.5 | 32.6 | -17.6 | 3.5 |
| 31 | 3 | 2 & 3 | YA | F | Prone | 3 | 0.708423 | 0.000009 | 11.5 | 15.9 | 31.7 | -19.4 | 3.2 |
| *33* | *3* | 2 & 3 | *UK* | *UK* | *Left side* | *3* | *0.707652* | *0.000013* | *7.5* | *16.7* | *25.6* | *-18* | *4* |
| 35 | 3 | 2 & 3 | YA | M | Prone | 4 | 0.709139 | 0.000013 | 10.9 | 13.9 | 32.8 | -21.2 | 3.5 |
| 36 | 3 | 2 & 3 | YA | M | No info | 3 |  |  | 10.3 | 15.2 | 31.6 | -20.2 | 3.6 |
| *37* | *3* | 2 & 3 | *MA-OA* | *F* | *Supine* | *3* | *0.70844* | *0.000012* | *5.3* | *14.3* | *17.9* | *-18.5* | *3.9* |
| 40 | 3 | 2 & 3 | OA | M | No info | 4 |  |  | 10.7 | 18.4 | 31.7 | -15.3 | 3.5 |
| 41.1 | 3 | 2 & 3 | OA | F | Prone | 3 | 0.708659 | 0.000014 | 14 | 17.8 | 40.7 | -15.7 | 3.4 |
| 42 | 3 | 2 & 3 | OA | F | Right side | 4 |  |  | 10.5 | 17.1 | 31.7 | -16.3 | 3.5 |
| 43 | 3 | 2 & 3 | OA | M? | No info | 4 |  |  | 12.8 | 15.9 | 38.1 | -18 | 3.5 |
| 44 | 3 | 2 & 3 | UK | UK | Flexed | 4 |  |  | 15.1 | 17.7 | 44.2 | -15.8 | 3.4 |
| *491* | *3* | 2 & 3 | *UK* | *UK* | *No info* | *4* |  |  | *2.8* | *16.4* | *14.6* | *-21.4* | *6.1* |

Legend:

^a^YA=Young adult (17-34 years), MA=Mid adult (35-49 years), OA=Old adult (50+) and UK=Adult with unknown age

^b^ M=Male, F=Female and UK=Adult of unknown sex.

^c^ Burial position.

^d^ Wealth: 1 = Greater than 10 artefacts, 2 = 6 – 10 artefacts, 3 = 1 – 5 artefacts, 4 = No artefacts. (Note: Multiple necklace units are combined as one artefact in this count except in cases where units are made from different materials or where it has been noted that they made up more than one necklace). Data from Duff 1977, List compiled by Sally Burage (Emeritus Curator Canterbury Museum).
