# Supplementary material for: The First New Zealanders? An Alternative Interpretation of Stable Isotope Data from Wairau Bar, New Zealand
Source: PLoS One. 2015 Oct 28;10(10):e0135214. doi: 10.1371/journal.pone.0135214 (PMC4624984; doi:10.1371/journal.pone.0135214)
Supplement: S2 Table — (DOCX) [file pone.0135214.s002.docx]

S2 Table. The results of stepwise model selection using both AIC and BIC criterions.

| **Response Variable** | **Model Steps** | **Covariates** | **AIC** | **BIC** |
| --- | --- | --- | --- | --- |
|  |  |  |  |  |
| δ^15^N (‰) | **Model A** |  |  |  |
|  | **1** | Kinaston Group, Age, Sex, Grave Goods | 26.35 | 31.94 |
|  | **2** | Kinaston Group, Age, Sex | 24.5 | 28.93 |
|  | **3** | Age, Sex | 22.6 | 25.88 |
|  | **4** | Sex | 21.4 | 23.61 |
| δ^15^N (‰) | **Model B** |  |  |  |
|  | **1** | Duff Group, Age, Sex, Grave Goods | 26.49 | 31.81 |
|  | **2** | Duff Group, Age, Sex | 24.56 | 28.87 |
|  | **3** | Age, Sex | 22.6 | 25.88 |
|  | **4** | Sex | 21.4 | 23.61 |
|  |  |  |  |  |
|  | **Model C** |  |  |  |
|  | **1** | Age, Sex, Grave Goods | 24.58 | 28.94 |
|  | **2** | Age, Sex | 22.6 | 25.88 |
|  | **3** | Sex | 21.43 | 23.61 |
|  |  |  |  |  |
| δ^13^C (‰) | **Model D** |  |  |  |
|  | **1** | Kinaston Group, Age, Sex, Grave Goods | 27.4 | 32.86 |
|  | **2** | Kinaston Group, Sex, Grave Goods | 25.43 | 29.8 |
|  | **3** | Kinaston Group, Grave Goods | 23.46 | 26.73 |
| δ^13^C (‰) | **Model E** |  |  |  |
|  | **1** | Duff Group, Age, Sex, Grave Goods | 28.2 | 33.65 |
|  | **2** | Duff Group, Sex, Grave Goods | 26.2 | 30.58 |
|  | **3** | Duff Group, Grave Goods | 25.54 | 27.82 |
| δ^13^C (‰) | **Model F** |  |  |  |
|  | **1** | Age, Sex, Grave Goods | 28.63 | 32.99 |
|  | **2** | Age, Sex | 26.7 | 29.94 |
|  | ***3*** | Sex | 25.28 | 27.46 |
|  |  |  |  |  |
